# Arthrogenic muscle inhibition after anterior cruciate ligament injury: Injured and uninjured limb recovery over time

**DOI:** 10.3389/fspor.2023.1143376

**Published:** 2023-03-21

**Authors:** April L. McPherson, Nathan D. Schilaty, Sarah Anderson, Takashi Nagai, Nathaniel A. Bates

**Affiliations:** ^1^ Emory Sport Performance and Research Center, Emory University, Flowery Branch, GA, United States; ^2^ Department of Orthopaedics, The Ohio State University, Columbus, OH, United States; ^3^ Department of Physiology & Biomedical Engineering, Mayo Clinic, Rochester, MN, United States; ^4^ Department of Physical Medicine & Rehabilitation, Rochester, MN, United States; ^5^ Department of Orthopedic Surgery, Mayo Clinic, Rochester, MN, United States; ^6^ Sports Medicine Center, Mayo Clinic, Rochester, MN, United States; ^7^ Department of Neurosurgery & Brain Repair, University of South Florida, Tampa, FL, United States; ^8^ Department of Medical Engineering, University of South Florida, Tampa, FL, United States; ^9^ United States Army Research Institute of Environmental Medicine, Natick, MA, United States

**Keywords:** ACL reconstruction (ACLR), motor unit (MU), motor unit recruitment, quadriceps, hamstrings, motor control

## Abstract

**Introduction:**

It is well documented that marked weakness of the quadriceps is present after knee joint injury. This joint trauma induces a presynaptic reflex inhibition of musculature surrounding the joint, termed arthrogenic muscle inhibition (AMI). The extent to which anterior cruciate ligament (ACL) injury affects thigh musculature motor unit activity, which may affect restoration of thigh muscle strength after injury, is undetermined.

**Methods:**

A randomized protocol of knee flexion and extension isometric contractions (10%–50% maximal voluntary isometric contraction) were performed for each leg on 54 subjects with electromyography array electrodes placed on the vastus medialis, vastus lateralis, semitendinosus, and biceps femoris. Longitudinal assessments for motor unit recruitment and average firing rate were acquired at 6-month intervals for 1 year post ACL injury.

**Results:**

The ACL-injured population demonstrated smaller quadriceps and hamstrings motor unit size (assessed *via* motor unit action potential peak-to-peak amplitude) and altered firing rate activity in both injured and uninjured limbs compared to healthy controls. Motor unit activity remained altered compared to healthy controls at 12 months post ACL reconstruction (ACLR).

**Discussion:**

Motor unit activity was altered after ACLR up to 12 months post-surgery. Further research is warranted to optimize rehabilitation interventions that adequately address altered motor unit activity and improve safety and success with return to sport after ACLR. In the interim, evidence based clinical reasoning with a focus on development of muscular strength and power capacity should be the impetus behind rehabilitation programming to address motor control deficits.

## Introduction

Thigh strength is vital for deceleration capacity in athletes, especially those in level I and II sports (1, 2). Anterior cruciate ligament (ACL) injuries are highest in athletes who participate in such sports that involve high volumes of acceleration, deceleration, and change of direction (1, 3). Naturally, rehabilitation for return to sport (RTS) after ACL reconstruction (ACLR) should ensure that athletes redevelop these capacities to reduce risk of reinjury, which has been reported as high as 33% within two years of RTS (4, 5). However, studies consistently report thigh muscle strength deficits at RTS (6), despite many RTS testing batteries requiring 85%–90% limb symmetry in thigh muscle strength and power (7).

It is well documented that marked weakness of the quadriceps is present after knee joint injury (8). This joint trauma induces a presynaptic reflex inhibition of the musculature surrounding the knee joint, termed arthrogenic muscle inhibition (AMI) (9–11). It is well established that AMI persists after ACL injury and during rehabilitation after ACLR; however, different mechanisms that contribute to AMI and their effects continue to be explored. It is well known that deficits exist and persist in central neural activation after ACLR and beyond RTS (8, 12). Previous studies have demonstrated acute and long-term changes in both central and peripheral mechanisms of inhibition, including altered electrocortical brain activity, increased excitability of the spinal-reflex pathways and reduced excitability of corticospinal pathways (13–15), and central activation failure (14, 15). Further, with the hypothesized presynaptic reflex inhibition of the thigh musculature and known central activation alterations post ACL-injury, it would follow that changes of motor control are evident in analysis of the electrical activity of the thigh muscles (12). These neural deficits may contribute to aberrant motor patterns that could contribute to future ACL injury risk, even though patients are expected to achieve 85%–90% limb symmetry on functional tasks necessary to return to sport. However, to date, there are limited methods used to assess AMI in the ACL-injured population, which hinders clinicians' and researchers' abilities to assess, quantify, and adequately address these deficits in a rehabilitation setting. Given the current suboptimal RTS outcomes after ACLR that persist up to one year after ACL injury (16–22) and the high rates of reinjury (4, 5, 23), research methods to improve understanding and the effects of AMI are warranted.

Muscle force generation is a product of both motor unit (MU) rate coding and quantity of MU recruitment, and it is well established that there an inverse relationship exists between MU rate coding and MU recruitment threshold (24). Previous work utilizing decomposed electromyography (dEMG) techniques has demonstrated altered MU activity in an ACL-injured population (25) Specifically, ACL-injured subjects demonstrated decreased size of recruited MUs and decreased rate coding compared to healthy controls (26); however, differences between the injured and uninjured limb were not assessed. Previous work has demonstrated the both the injured and uninjured limb have altered quadriceps activation after an ACL injury (7); thus, investigation of injured and uninjured limb motor control relative to healthy subjects is warranted.

The purpose of this study was to further describe thigh musculature motor unit (MU) activity in individuals who sustained ACL injury and completed standard rehabilitation and clinical care after ACL injury. Specifically, electromyography signals were captured and decomposed to characterize recruited MU size [assessed *via* MU action potential peak-to-peak amplitude (27)], time of recruitment, and average firing rate of uninjured and injured limbs within ACL-injured subjects over time throughout recovery, with additional comparison to healthy control subjects. It was hypothesized that quadriceps and hamstrings MU action potential (MUAP) peak-to-peak amplitude would be decreased from that of healthy controls, particularly acutely after injury, and MU average firing rate (AvgFR) would be lower than healthy controls for both the injured and uninjured limbs.

## Materials and methods

The Mayo Clinic Institutional Review Board approved this study (16-010600). Fifty-four subjects were recruited (see Table 1 for demographics) and completed written informed consent compliant with the latest revision of the Declaration of Helsinki. ACL-injured subjects were recruited either prior to surgery (data capture the day prior to ACLR; *n* = 24; “ACL Pre-Surg”) or at 6 months post-surgery (±1 month; *n* = 6). Of the ACL-injured subjects, 7 (23%) had experienced a second ACL tear prior to recruitment, a percentage consistent with existing literature (28). Subjects (*n* = 3) with bilateral ACL injuries were excluded from analysis. The ACL-injured subjects were followed longitudinally for testing intervals of 6 months (±1 month), which could include 6 months post-surgery (total *n* = 14; “ACLR 6mo”) and/or 12 months post-surgery (total *n* = 12; “ACLR 12mo”). Control subjects (*n* = 25,“CTRL”) were also recruited and were followed longitudinally for 6 months after the initial visit. Among the ACL-injured subjects, 73% received a bone-patellar-bone autograft, 24% received a hamstring autograft, and 3% received an allograft. Control subjects (*n* = 25, “CTRL”) were also recruited and followed longitudinally for 6 months after the initial visit. From initial recruitment, the overall attrition rate was 26%. Subject inclusion criteria were healthy, active individuals between the ages of 14–25 years old. Exclusion criteria included previous lower extremity injury or surgery in the past 6 months (other than ACL), neurological disorders, paralysis, neuromuscular disease, cardiovascular disease, exercise-induced injury, asthma, and pregnancy.

**Table 1:** Population demographics (divided by sex and by group).

		Height (cm)	Mass (kg)	Age (yrs)	ACL-Injured (*n*)	Non-contact Mechanism of Injury (*n*)
**Sex**	**M (*n* = 24)**	180.3 ± 5.6	81.6 ± 13.8	19.4 ± 2.9	13	10 (77%)
	**F (*n* = 32)**	169.0 ± 7.2	66.4 ± 12.6	18.7 ± 3.2	18	14 (78%)
	**Significance**	**0.001**	**0.001**	0.397	1.000	1.000
**Group**	**Control (*n* = 25)**	173.9 ± 8.5	67.6 ± 13.4	18.8 ± 3.1	—	—
	**ACL-Injured (*n* = 31)**	173.8 ± 8.8	77.2 ± 15.1	19.1 ± 3.1	—	—
	**Significance**	0.948	**0.016**	0.726	—	—

All data collections were performed with the subjects positioned in a dynamometer (HumacNORM; CSMi, Stoughton, MA, United States). A custom load cell apparatus (MLP-300; Transducer Techniques, Temecula, CA, United States) was affixed to the dynamometer torque arm to measure the subjects' force production required for the EMG decomposition software (*dEMG Analysis*; Delsys, Natick, MA, United States) (26, 29, 30). For isometric knee extension testing, subjects were seated with their knee flexed to 80° (0° = full extension). Subjects were secured with straps at the shoulder and waist to minimize whole body movement during testing. Surface 5-pin dEMG electrodes (Bagnoli; Delsys, Natick, MA, United States) were placed on the muscle belly of both the vastus medialis (VM) and vastus lateralis (VL) muscles. Pairwise subtraction of voltages at the five detection surfaces was used to derive multi-channel sEMG signals. For isometric knee flexion testing, subjects were positioned prone on the dynamometer with knee flexed to 30° (0° = full extension). 5-pin dEMG electrodes were placed on the muscle belly of the biceps femoris (BF) and semitendinosus (ST) muscles. Prior to electrode placement, the skin was shaved and cleansed with an alcohol swab to ensure adequate skin-electrode contact. All electrodes were placed according to Surface Electromyography for the Non-Invasive Assessment of Muscles (SENIAM) standards (31).

### Testing protocol

Three isometric knee flexion and extension maximal voluntary isometric contractions (MVICs) were performed first to determine 10%, 25%, 35% and 50% MVIC contraction levels for each leg. Subjects were verbally encouraged to push as hard as possible for three seconds and the computer monitor was visible during the MVIC trials for visual feedback and motivation. A randomized protocol was generated for each subject to determine test order of limb side (right vs. left), muscle group (hamstring vs. quadriceps), and order of trials (10%–50% MVIC). All trials were repeated twice to ensure adequate MU data capture. Each trial consisted of following a trapezoidal waveform (three second ramp up, ten second sustained contraction at designated %MVIC, three second ramp down). Subject were instructed to follow the trapezoidal waveform displayed *via* real-time visual feedback on a monitor.

### EMG signal decomposition

MUAP peak-to-peak amplitude and average firing rates (AvgFR) were captured through sEMG. MUAP peak-to-peak amplitude has been used in the literature as a representative for MU size (27, 32). The analog sEMG channels were band-pass filtered with cut-off frequencies of 20 and 1750Hz. Each channel was over-sampled at 20 kHz to avoid introduction of significant phase skew across channels. The sEMG signals were digitally filtered using a high-pass filter with a cutoff frequency of 50 Hz before decomposition (33). The signal decomposition algorithm first extracted action potential “templates” of as many MUAP trains as possible from the input sEMG signal. The algorithm then searched for signal regions where the extracted MUAP train templates were superimposed with other identified MUAP trains or with unidentified action potentials. The algorithm takes both constructive and destructive interference effects into account when analyzing such superpositions. Moreover, the algorithm requires that the unidentified action potentials account for less than 25% of the signal energy at the firing locations of the decomposed MUAP trains (33).

In order to verify the decomposed signal, the algorithm performed a Decompose-Synthesize-Decompose-Compare test (25, 33). The original signal was decomposed, as described in the preceding paragraph. Then, white noise with a root mean square error value equivalent to the residual of the non-decomposed signal was added to the decomposed signal and synthesized. This synthesized signal was then decomposed again, as described above, and compared to the original signal decomposition. Only MUs with an accuracy of ≥90% were included in analysis for the current study. In addition to internal validation by the development group, the decomposition algorithm has been externally validated against spike triggered averaging technique and further details of the methodology can be found elsewhere (33, 34). This technique has been able to determine differences between both healthy and pathological conditions (26, 35).

### Rehabilitation protocol

Each subject followed the standard of care protocol at the healthcare institution, and the treatment guidelines of their respective physical therapist. All ACL-injured subjects were permitted to return to sport between 9 and 11 months once standard of care targets of the medical care team, including the treating orthopedic surgeon and respective physical therapist were achieved.

### Data analysis

All statistical analyses were performed with *JMP Pro 16* (SAS Institute Inc., Cary, NC, United States). As MU strategies are non-linear (36, 37), *log* MUAP peak-to-peak amplitude and *cube root* Recruitment Threshold transformations were utilized to provide parametric data for linear regressions. MU data were normalized to force and mass for statistical analysis, when appropriate. Additionally, we calculated an interaction these variables (AvgFR * MUAP * Recruitment Threshold) to assess the overall contribution of each MU factors to muscle activity. Descriptive statistics were calculated for both sex and group *via* Student's *t*-test and Fisher's Exact test and reported as mean (SD) or n values, as appropriate (Table 1). MUAP peak-to-peak amplitude and AvgFR were compared between groups with standard least squares regression and least square means ANOVA. Tukey's HSD post-hoc comparisons were utilized when appropriate. Significance was set *a priori* at *p* < 0.05.

## Results

For all groups, a total of 37,827 MUs were identified and analyzed. The decomposition methodology identified 18,097 hamstring MUs and 19,730 quadriceps MUs. Within the quadriceps MUs, 10,654 MUs were VL and 9,076 were VM. For the hamstring MUs, 8,619 MUs were from BF and 9,478 were ST. 9,762 MUs were classified as injured limbs and 28,065 were uninjured limbs, which included control subjects' limbs.

### MUAP peak-to-peak amplitude

**Figure 1:**
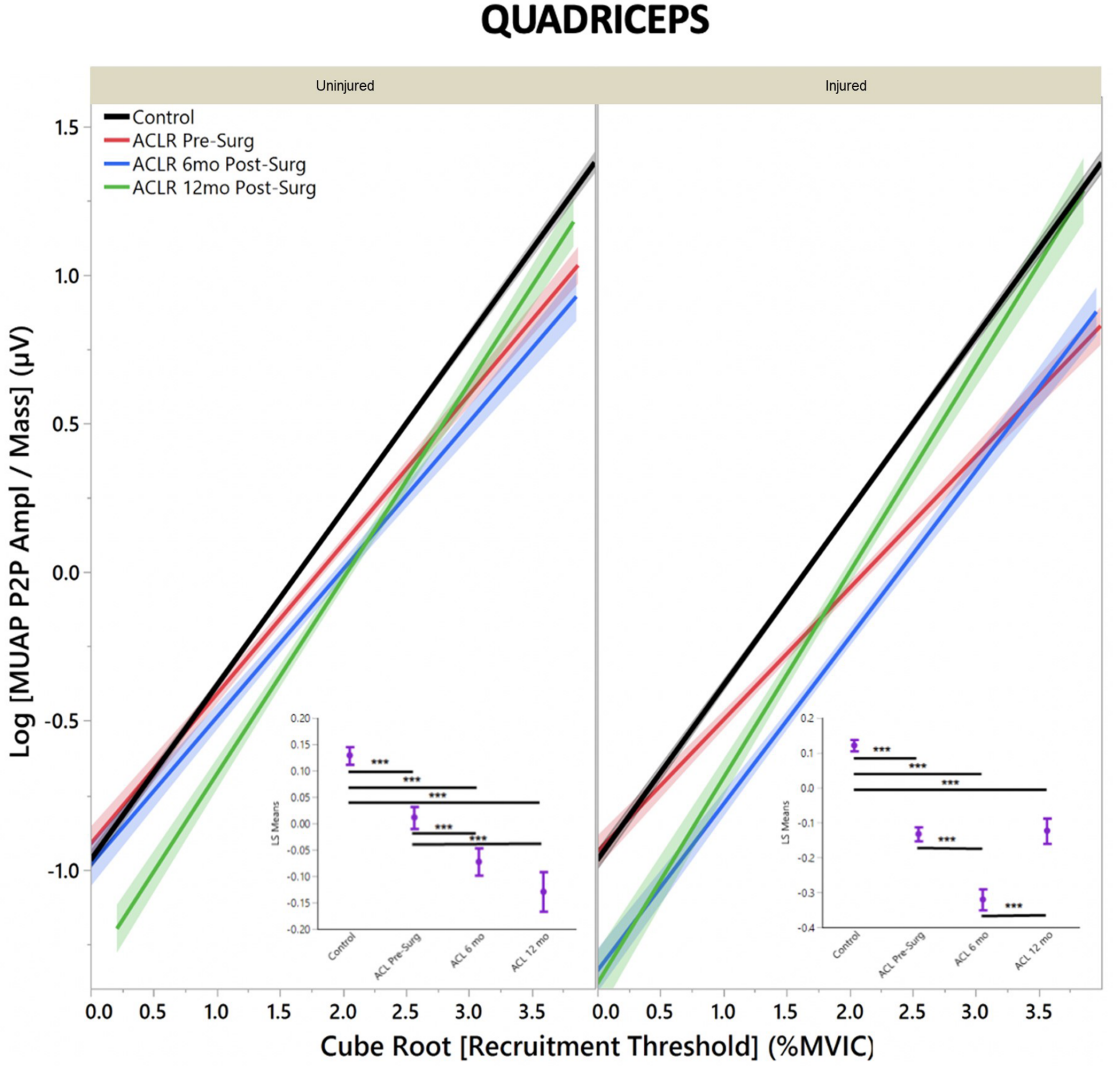
Quadriceps motor unit action potential by group for uninjured and injured limbs. MUAP peak-to-peak amplitude represents motor unit size. (**Insets**) Least means square ANOVA demonstrates significant differences of MUAP between groups. (* denotes *p* < 0.05; *** denotes *p* < 0.001; line shading and error bars denote 95% confidence intervals of the mean.)

**Figure 2:**
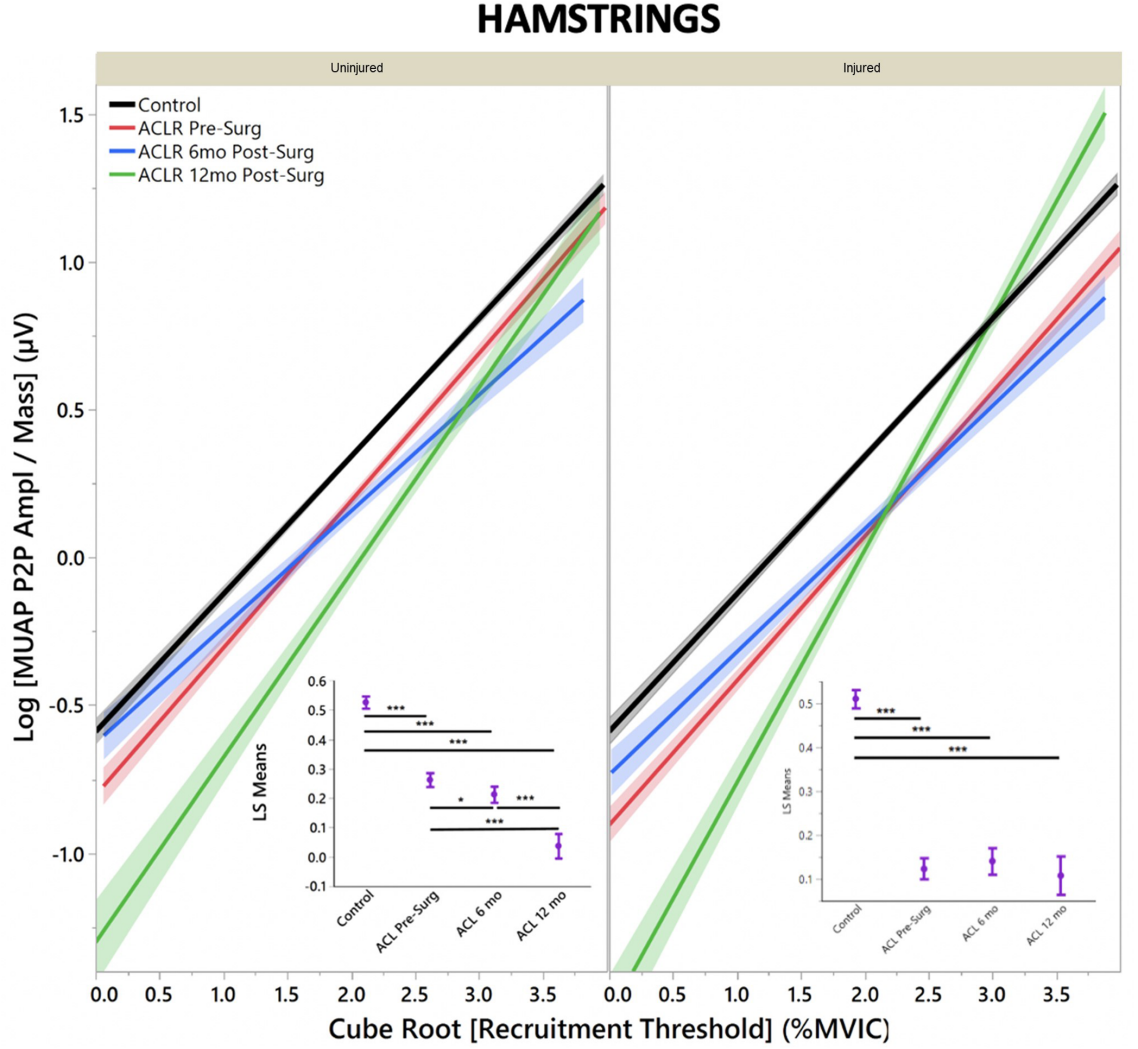
Hamstring motor unit action potential by group for uninjured and injured limbs. MUAP Peak to Peak amplitude represents motor unit size. (**Insets**) Least means square ANOVA demonstrates significant differences of MUAP between groups. (* denotes *p* < 0.05; *** denotes *p* < 0.001; line shading and error bars denote 95% confidence intervals of the mean.).

The regression models of MUAP peak-to-peak amplitude by recruitment threshold demonstrated that both quadriceps (Figure 1) and hamstrings (Figure 2) in uninjured and injured limbs of ACL-injured have lower MUAP peak-to-peak amplitude across all time points when compared with CTRLs (*R*^2^ Quad^un^*^ ^*= 0.42; *R*^2^ Quad^inj^*^ ^*=^ ^0.44; *R*^2^ Hams^un ^= 0.36; *R*^2^ Hams^inj^ = 0.40).

For ACL-injured subjects, quadriceps MUAP peak-to-peak amplitude for a given recruitment threshold did not change from pre-surgery to 6 months post-ACLR in the uninjured limb (Figure 1, inset; LS mean = −0.04 vs. −0.07, respectively; *p* = 0.26). However, MUAP peak-to-peak amplitude decreased from 6 months post-ACLR to 12 months post-ACLR (LS mean = −0.15; *p* = 0.03). For the injured limb, MUAP peak-to-peak amplitude decreased from pre-surgery to 6 months post-ACLR (Figure 1, inset; LS mean = −0.16 vs. −0.33, respectively; *p* < 0.001). However, in contrast to the uninjured limb, MUAP peak-to-peak amplitude increased between 6 months post-ACLR and 12 months post-ACLR (LS mean = −0.33 vs. −0.13, respectively; *p* < 0.001).

Uninjured limb hamstrings musculature demonstrated a decrease in MUAP peak-to-peak amplitude across all timepoints from pre-surgery to 12 months post-ACLR (Figure 2, inset; LS mean = 0.25 vs. 0.214 vs. 0.03, respectively; *p* < 0.001). Injured limb hamstrings musculature did not demonstrate any significant differences between time pre-surgery and 6 months post-ACLR (Figure 2, inset; LS mean = 0.14 vs. 0.13; *p* = 0.98), but significantly between 6 months post-ACLR and 12 months post-ACLR, as well as compared to pre-surgery for MUAP peak-to-peak amplitudes (LS mean = 0.13 vs. 0.21, respectively; *p* ≤ 0.05).

### MU average firing rate

**Figure 3:**
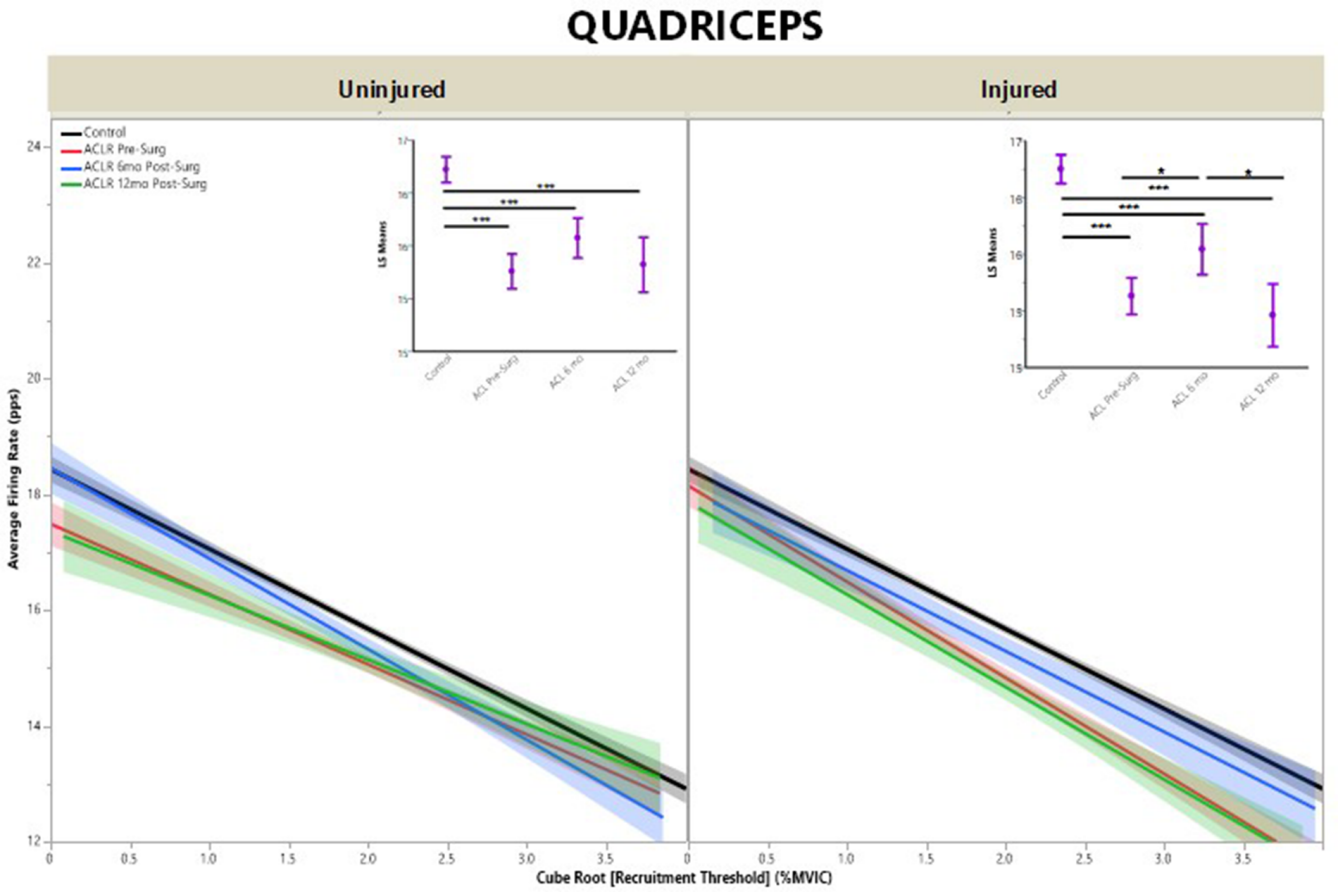
Quadriceps average firing rate by group for uninjured and injured limbs. Average firing rate represents motor unit firing frequency. (**Insets**) Least means square ANOVA demonstrates significant differences of MUAP between groups. (* denotes *p* < 0.05; *** denotes *p* < 0.001; line shading and error bars denote 95% confidence intervals of the mean.).

**Figure 4:**
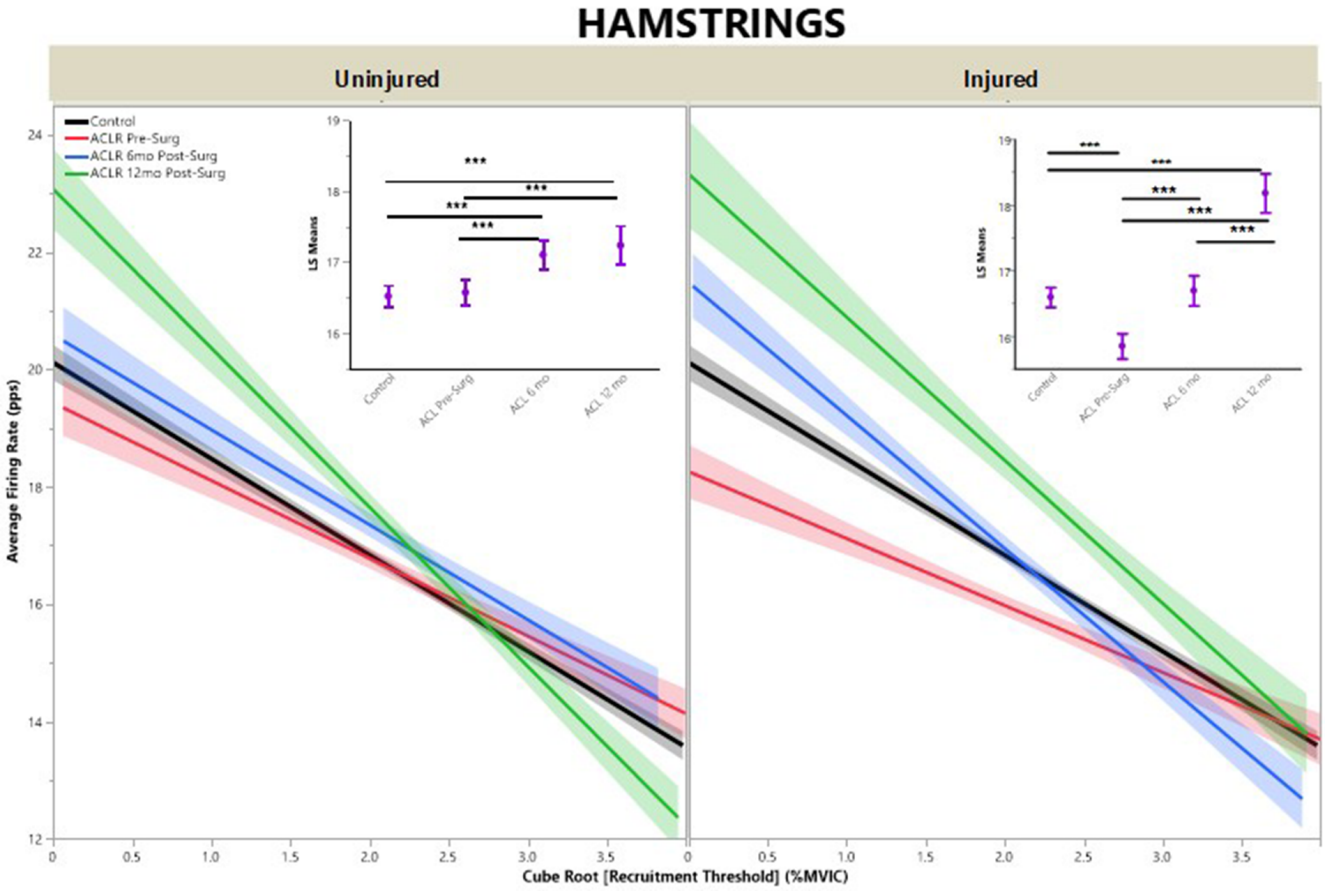
Hamstrings average firing rate by group for uninjured and injured limbs. Average firing rate represents motor unit firing frequency. (Insets) Least means square ANOVA demonstrates significant differences of MUAP between groups. (* denotes *p* < 0.05; *** denotes *p* < 0.001; line shading and error bars denote 95% confidence intervals of the mean.).

For AvgFR by recruitment threshold, the regression models demonstrated that quadriceps (Figure 3) had lower AvgFR across all timepoints when compared with CTRLs for both injured and uninjured limbs and higher AvgFR across all timepoints for the hamstrings (Figure 4), except for the injured limb at pre-surgery (*R*^2^ Quad^un ^= 0.09; *R*^2^ Quad^inj ^=^ ^0.11; *R*^2^ Hams^un ^= 0.11; *R*^2^ Hams^inj^ = 0.13).

For ACL-injured subjects, quadriceps AvgFR for a given recruitment threshold did not change between pre-surgery, 6 months post-ACLR, and 12 months post-ACLR in the uninjured limb (Figure 3, inset; LS mean = 15.3 vs. 15.6 vs. 15.3, respectively; *p* ≥ 0.06). For the injured limb, AvgFR increased from pre-surgery to 6 months post-ACLR (Figure 3, inset; LS mean = 15.1 vs. 15.5, respectively; *p* = 0.02), but returned back towards pre-surgery levels at the 12-month post-ACLR timepoint (LS mean = 14.5, *p* < 0.01).

Uninjured limb hamstrings musculature demonstrated increased AvgFR from pre-surgery to 6 months post-ACLR (Figure 4, inset; LS mean = 16.6 vs. 17.1, respectively; *p* < 0.001), but was no different at 12 months post-ACLR (LS mean = 17.2, *p* ≥ 0.87) from either pre-surgery or 6 months post-ACLR. Injured limb hamstrings musculature AvgFR increased across all three timepoints, from pre-surgery (LS mean = 15.9) to 6 months post-ACLR (LS mean = 16.7) to 12 months post-ACLR (LS mean = 18.2, *p* < 0.001).

### AvgFR * MUAP * Recruitment Threshold interaction

**Figure 5:**
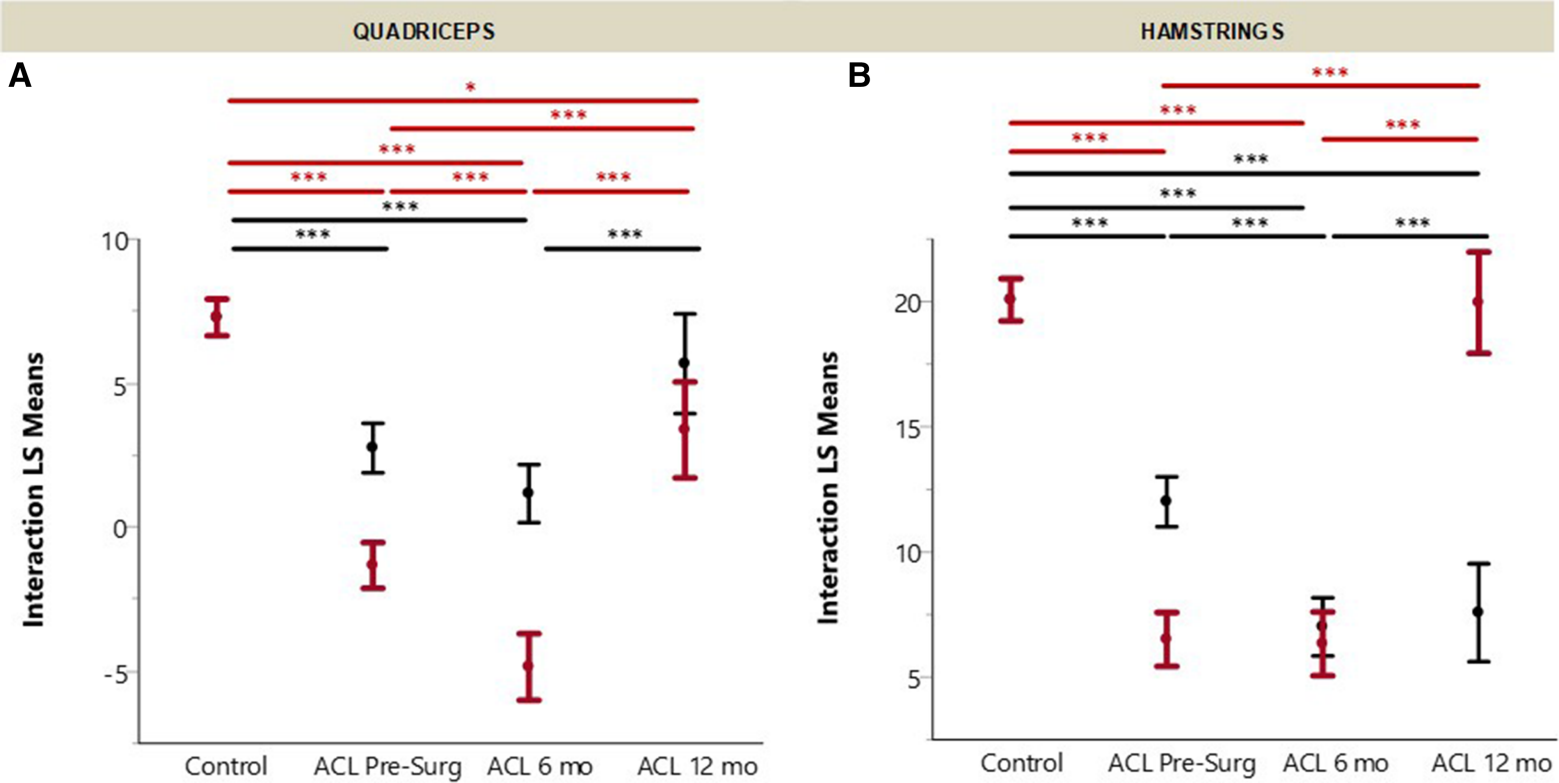
ACL recovery of (**A**) hamstrings and (**B**) quadriceps in the injuredand uninjured limbs. Recovery measured by multiplication of average firing rate (AvgFR), log of MUAP divided by mass [log(MUAP/mass)], and recruitment threshold [CubeRoot(RecThr)]. (Error bars denote 95% confidence intervals of the mean.).

For the quadriceps (Figure 5A), the interaction term was lower in the uninjured limb for pre-surgery and 6 months post-ACLR (*p* < 0.001), but no at 12 months post-ACLR (*p* = 0.68) compared to controls. Pre-surgery was not different from 6 months post-surgery or from 12 months post-ACLR (*p* ≥ 0.06), but 6 months post-surgery was significantly lower than 12 months post-ACLR (*p* < 0.01). For the injured limb, all ACL-injured groups were significantly lower than controls (*p* < 0.01), and all three ACL-injured groups were significantly different from each other (*p* < 0.01).

For the hamstrings (Figure 5B), the interaction term was lower in the uninjured limb for all ACL-injured groups compared to controls (*p* < 0.01). Pre-surgery was higher than both 6 and 12 months post-ACLR (*p* < 0.01), but 6 and 12 months post-ACLR were not significantly different (*p* = 0.99). For the injured limb hamstrings, both pre-surgery and 6 months post-ACLR were lower than controls (*p* < 0.01), but 12 months was not different than controls (*p* = 1.00). Pre-surgery and 6 months post-ACLR were not different from each other (*p* = 1.00), but both groups were lower than 12 months post-ACLR (*p* < 0.01).

## Discussion

This is the first study that has quantified MU activity of injured and uninjured limbs post ACL injury. This study confirms an ACL-injured population demonstrates smaller quadriceps and hamstrings MU size in both injured and uninjured limbs compared to healthy CTRLs. In addition, ACL-injured subjects demonstrated reduced AvgFR compared to healthy controls for the quadriceps musculature but mostly increased AvgFR for hamstrings musculature in both the injured and uninjured limbs. Together, these findings partially support the hypotheses of this study. Consistent with previous findings in this cohort, MU activity did not appear to fully recover at 12 months post-ACLR compared to healthy control subjects (26). These findings provide important insight for efforts to improve rehabilitation in athletes with ACLR who aim to return to level I or II sports. These findings provide important insight into neuromuscular and motor control deficits that could persist in athletes with ACLR up to 12 months postoperatively.

The finding that both quadriceps and hamstrings musculature MUAP amplitude is reduced in both injured and uninjured limbs compared to controls suggests that post-ACL injury, smaller MUs are favored for recruitment and utilization to achieve a targeted force output. We speculate that this reduced MU size may be compensated for in the hamstrings musculature by the increased AvgFR observed in the ACL-injured subjects since it is known that in healthy musculature, neural activity adapts varying strategies *via* AvgFR and MU recruitment to achieve a desired force output (25, 38). However, this similar effect was not observed for the quadriceps musculature in either the injured or uninjured limb. This supports the previous finding that quadriceps activation is bilaterally impaired, despite a unilateral ACL injury (39). This may be correlated with the muscle atrophy that clinicians have observed with the quadriceps musculature post-ACL injury and warrants further exploration in future research (40).

All together, these data suggest that at 12 months post-ACLR small MUs do not function like those of healthy controls. It appears that fine motor control may be altered, as demonstrated by differential small MU recruitment and firing rate patterns in the ACL-injured subjects across timepoints compared to healthy controls. This can further be seen when looking at recovery as a whole, through the lens of the interaction between the three main MU characteristics that drive muscle output: MUAP peak-to-peak amplitude, recruitment threshold, and AvgFR (Figure 5). For both the quadriceps and hamstrings, neither the injured or uninjured limb reach healthy control subjects' values with the exception of the injured limb hamstrings, which suggests and further supports that the traumatic knee injury affects not only the involved limb but may also have a crossover effect (41). These findings suggest smaller MUs in conjunction with altered firing rate is a strategy the central nervous system may adopt after an ACL injury to achieve a desired result. Together, these altered MU strategies may provide insight into the neural mechanisms that contribute to the observed deficits associated with AMI.

It is interesting to note that for both the quadriceps and hamstrings muscles at 6 months post-ACLR, both injured and uninjured limbs' regression lines follow a similar slope to the healthy controls (Figures 1, 2), which may suggest that during this period of heavily focused rehabilitation, the subjects may begin to reattain motor control more similar, yet depressed, to healthy controls. However, by the 12-month post-ACLR timepoint, there is yet another shift in the MU size trend away from that of the healthy controls. Interestingly, this trend is also observed in the injured limb at the 12-month post-ACLR timepoint for both quadriceps and hamstrings musculature, where quadriceps AvgFR returns to near pre-surgery levels further away from the control level and hamstrings AvgFR increases away from the control level. Previous studies have similarly demonstrated altered neural activation and motor control deficits at 12 months post-ACLR and beyond, *via* different methodologies (12, 14, 15). The findings of this study, in combination with previous investigations that have identified altered motor control, may suggest that rehabilitation should be extended until motor control, both central and specifically of the thigh musculature, returns and is more similar to motor control of healthy subjects (26, 42).

Future research should investigate the effects of targeted interventions in individuals after ACLR who may demonstrate factors associated with AMI. This work could help optimize interventions to address lingering motor control deficits despite current recommended clinical practice. Interestingly, strength training literature has shown that training with heavy loads (>80% of 1 repetition maximum) increased neural drive; in addition, intent to move weight quickly and ballistic power training methods have helped increase rate of force development by lowering MU recruitment thresholds (43). Such interventions could be considered to determine utility to address AMI in both ipsilateral and contralateral limbs after ACL injury. It is currently unknown if these changes in motor control ever fully resolve or return to the patterns observed in the healthy control subjects, as our longitudinal data collection stopped at 12 months post-ACLR. It may be possible that longer investigations are warranted to determine if interventions can fully restore normal motor control patterns after a traumatic knee injury.

Despite an extensive body of clinical research, RTS rates after ACLR are suboptimal (17–19, 23). In an updated systematic review by Ardern et al. [69 articles; *n* = 7,556 athletes (66% male); average age: 25 ± 3.2 years], the authors found only 65% of athletes returned to preinjury level of sport after ACLR; of those who previously participated in competitive sports, only 55% returned to the competitive level after ACLR (17). These rates were consistent with another study that reported 56% of young athletes (17.1 ± 2.4 years, *n* = 124) who planned to return to level I or level II sports returned to preinjury level after ACLR (21). Further, the body of evidence suggests ACL reinjury rates for those who RTS after ACLR are at least 20% (4, 5, 23) and individuals who RTS after ACLR are 15× more likely to sustain another ACL injury than controls (44, 45). The extent to which AMI affects these RTS and reinjury rates may be a missing link in current RTS assessments, potentially suggesting suboptimal management after ACLR. Evidence-based recommendations for RTS include evaluation of readiness through a battery of tests, including open tasks when possible, incorporating reactive decision making, and assessing psychological readiness (46). Standard clinical and rehabilitation practices often utilize peak torque for normalized comparisons of strength capacity and assessment of readiness for RTS. Inclusion of these values in combination with MU information could be additive to improve the current understanding of AMI and associated risk of ACL reinjury (both ipsilateral and contralateral limb).

Persistent motor deficits after ACLR, such as decreased strength and activation capacity, have been linked to an increased risk of reinjury (47, 48). Additionally, bilateral motor deficits after injury have negative repercussions for normative tests that use within-subject limb-to limb-comparisons. Strength decrements in the uninjured limb after ACLR can provide a false indication of readiness to RTS when using limb symmetry indices (LSIs) (49). Given the bilateral decrease in ACL-injured subjects’ MUAP peak amplitude in the current study compared to control subjects that was found up to 12 months post-ACLR, changes in the uninjured limb could contribute to an altered sense of physical readiness and a unsubstantiated quantification of achieving an 85%–90% LSI. These findings warrant considerations for rehabilitation programming and implications for further research.

The current study is not without limitations but could help to inform future investigations. Use of the HumacNORM dynamometer added additional noise to the dEMG signal; therefore, we had to use a custom load cell in place of the dynamometer to capture subjects' force output. Thus, we were only able to register force and could not accurately translate this information to joint torque. In addition, the isometric tasks utilized in the current study may not be representative of dynamic tasks required for rehabilitation and sport clearance. Thus, future studies could investigate differences between injured and uninjured limbs in more dynamic, athletically relevant tasks with the use of wireless dEMG sensors.

In conclusion, ACL-injured subjects demonstrated altered quadriceps and hamstrings MUAP activity in both the injured and uninjured limbs relative to healthy controls across multiple timepoints post injury. MUAP activity was different pre-operatively, post-ACLR, and persisted throughout rehabilitation up to 12 months post-operatively. Further research is warranted to determine if MUAP peak amplitude is reflective and consistent with other implications of AMI, and then to optimize rehabilitation interventions that address altered MU activity to improve RTS outcomes after ACLR. Evidence-based clinical reasoning with a focus on development of muscular strength and power capacity should be the impetus behind rehabilitation programming to address potential motor deficits.

## Data Availability

The datasets presented in this study can be found in online repositories. The names of the repository/repositories and accession number(s) can be found below: https://zenodo.org/record/7620333#.Y_Od2HbMK3A.

## References

[1] Harper DJ McBurnie AJ Santos TD Biomechanical and neuromuscular performance requirements of horizontal deceleration: a review with implications for random intermittent multi-directional sports. Sports Med. (2022) 52(10):2321–54. 10.1007/s40279-022-01693-0 35643876 PMC9474351

[2] Hefti E Müller W Jakob RP Stäubli HU. Evaluation of knee ligament injuries with the IKDC form. Knee Surg Sports Traumatol Arthrosc. (1993) 1(3-4):226–34. 10.1007/BF01560215 8536037

[3] Dodson CC Secrist ES Bhat SB Woods DP Deluca PF. Anterior cruciate ligament injuries in national football league athletes from 2010 to 2013: a descriptive epidemiology study. Orthop J Sports Med. (2016) 4(3). 10.1177/2325967116631949 PMC4780097 26998501

[4] Wiggins AJ Grandhi RK Schneider DK Stanfield D Webster KE Myer GD. Risk of secondary injury in younger athletes after anterior cruciate ligament reconstruction: a systematic review and meta-analysis. Am J Sports Med. (2016):1–16. 10.1177/0363546515621554 PMC5501245 26772611

[5] Wright RW Haas K Anderson J Anterior cruciate ligament reconstruction rehabilitation: MOON guidelines. Sports Health. (2014) 7(3):239–43. 10.1177/1941738113517855 PMC4482298 26131301

[6] Lepley LK. Deficits in quadriceps strength and patient-oriented outcomes at return to activity after ACL reconstruction: a review of the current literature. Sports Health. (2015) 7(3):231–8. 10.1177/1941738115578112 26131300 PMC4482305

[7] Grindem H Snyder-Mackler L Moksnes H Engebretsen L Risberg MA. Simple decision rules can reduce reinjury risk by 84% after ACL reconstruction: the Delaware-Oslo ACL cohort study. Br J Sports Med. (2016) 50(13):804–8. 10.1136/bjsports-2016-096031 27162233 PMC4912389

[8] Rice DA McNair PJ. Quadriceps arthrogenic muscle inhibition: neural mechanisms and treatment perspectives. Semin Arthritis Rheum. (2010) 40(3):250–66. 10.1016/j.semarthrit.2009.10.001 19954822

[9] Hart JM Pietrosimone B Hertel J Ingersoll CD. Quadriceps activation following knee injuries: a systematic review. J Athl Train. (2010) 45(1):87–97. 10.4085/1062-6050-45.1.87 20064053 PMC2808760

[10] Hopkins JT Ingersoll CD. Arthrogenic muscle inhibition: a limiting factor in joint rehabilitation. J Sport Rehabil. (2000) 9(2):135–59. 10.1123/jsr.9.2.135

[11] Pietrosimone BG Hopkins JT Ingersoll CD. Therapeutic modalities: the role of disinhibitory modalities in joint injury rehabilitation. Athl Ther Today. (2008) 13(6):2–5. 10.1123/att.13.6.2

[12] Pietrosimone B Lepley AS Kuenze C Arthrogenic muscle inhibition following anterior cruciate ligament injury. J Sport Rehabil. (2022) 31(6):1706. 10.1123/jsr.2021-0128 35168201

[13] Baumeister J Reinecke K Schubert M Weiß M. Altered electrocortical brain activity after ACL reconstruction during force control. J Orthop Res. (2011) 29(9):1383–9. 10.1002/jor.21380 21437965

[14] Rodriguez KM Palmieri-Smith RM Krishnan C. How does anterior cruciate ligament reconstruction affect the functioning of the brain and spinal cord? A systematic review with meta-analysis. J Sport Health Sci. (2020) 00:1–10. 10.1016/j.jshs.2020.07.005 PMC7987657 32707098

[15] Tayfur B Charuphongsa C Morrissey D Miller SC. Neuromuscular function of the knee joint following knee injuries: does it ever get back to normal? A systematic review with meta-analyses. Sports Med. (2021) 51(2):321–38. 10.1007/s40279-020-01386-6 33247378 PMC7846527

[16] Ardern CL Taylor NF Feller JA Webster KE. A systematic review of the psychological factors associated with returning to sport following injury. Br J Sports Med. (2012):1120–6. 23064083 10.1136/bjsports-2012-091203

[17] Ardern CL Taylor NF Feller JA Webster KE. Fifty-five per cent return to competitive sport following anterior cruciate ligament reconstruction surgery: an updated systematic review and meta-analysis including aspects of physical functioning and contextual factors. Br J Sports Med. (2014) 48(21):1543–52. 10.1136/bjsports-2013-093398 25157180

[18] Ardern CL Taylor NF Feller JA Webster KE. Return-to-sport outcomes at 2 to 7 years after anterior cruciate ligament reconstruction surgery. Am J Sports Med. (2012) 40(1):41–8. 10.1177/0363546511422999 21946441

[19] Ardern CL Webster KE Taylor NF Feller JA. Return to sport following anterior cruciate ligament reconstruction surgery: a systematic review and meta-analysis of the state of play. Br J Sports Med. (2011) 45(7):596–606. 10.1136/bjsm.2010.076364 21398310

[20] Hettrich CM Dunn WR Reinke EK Spindler KP. The rate of subsequent surgery and predictors after anterior cruciate ligament reconstruction: two- and 6-year follow-up results from a multicenter cohort. Am J Sports Med. (2013) 41(7):1534–40. 10.1177/0363546513490277 23722056 PMC4195486

[21] Ithurburn MP Altenburger AR Thomas S Hewett TE Paterno Mv Schmitt LC. Young athletes after ACL reconstruction with quadriceps strength asymmetry at the time of return-to-sport demonstrate decreased knee function 1 year later. Knee Surg Sports Traumatol Arthrosc. (2017):0123456789. 10.1007/s00167-017-4678-4 28918506

[22] Ithurburn MP Paterno Mv Ford KR Hewett TE Schmitt LC. Young athletes with quadriceps femoris strength asymmetry at return to sport after anterior cruciate ligament reconstruction demonstrate asymmetric single-leg drop-landing mechanics. Am J Sports Med. (2015) 43(11):2727–37. 10.1177/0363546515602016 26359376

[23] Allen MM Pareek A Krych AJ Are female soccer players at an increased risk of second anterior cruciate ligament injury compared with their athletic peers? Am J Sports Med. (2016) 44(10):2492–8. 10.1177/0363546516648439 27261476

[24] Maffiuletti NA Aagaard P Blazevich AJ Folland J Tillin N Duchateau J. Rate of force development: physiological and methodological considerations. Eur J Appl Physiol. (2016) 116(6):1091–116. 10.1007/s00421-016-3346-6 26941023 PMC4875063

[25] de Luca CJ Hostage EC. Relationship between firing rate and recruitment threshold of motoneurons in voluntary isometric contractions. J Neurophysiol. (2010) 104(2):1034–46. 10.1152/jn.01018.2009 20554838 PMC2934917

[26] Schilaty ND McPherson AL Nagai T Bates NA. Arthrogenic muscle inhibition manifests in thigh musculature motor unit characteristics after anterior cruciate ligament injury. Eur J Sport Sci. (2022). 35306977 10.1080/17461391.2022.2056520 PMC9626399

[27] Hu X Rymer WZ Suresh NL. Motor unit pool organization examined via spike-triggered averaging of the surface electromyogram. J Neurophysiol. (2013) 110(5):1205–20. 10.1152/jn.00301.2012 23699053 PMC4073930

[28] Schilaty ND Nagelli Cv Bates NA Incidence of second anterior cruciate ligament tears and identification of associated risk factors from 2001 to 2010 using a geographic database. Orthop J Sports Med. (2017) 5(8):1–8. 10.1177/2325967117724196 PMC5564962 28840155

[29] de Luca CJ Contessa P. Biomechanical benefits of the onion-skin motor unit control scheme. J Biomech. (2015) 48(2):195–203. 10.1016/j.jbiomech.2014.12.003 25527890 PMC4295621

[30] de Luca CJ Contessa P. Hierarchical control of motor units in voluntary contractions. J Neurophysiol. (2012) 107(1):178–95. 10.1152/jn.00961.2010 21975447 PMC3349682

[31] Hermens HJ Freriks B Disselhorst-Klug C Rau G. Development of recommendations for SEMG sensors and sensor placement procedures. J Electromyogr Kinesiol. (2000) 10(5):361–74. 10.1016/S1050-6411(00)00027-4 11018445

[32] Hu X Suresh AK Rymer WZ Suresh NL. Altered motor unit discharge patterns in paretic muscles of stroke survivors assessed using surface electromyography. J Neural Eng. (2016) 13(4):1–10. 10.1088/1741-2560/13/4/046025 PMC5439147 27432656

[33] Nawab SH Chang SS de Luca CJ. High-yield decomposition of surface EMG signals. Clin Neurophysiol. (2010) 121(10):1602–15. 10.1016/j.clinph.2009.11.092 20430694 PMC2932793

[34] de Luca CJ Chang SS Roy SH Kline JC Nawab SH. Decomposition of surface EMG signals from cyclic dynamic contractions. J Neurophysiol. (2015) 113(6):1941–51. 10.1152/jn.00555.2014 25540220 PMC4359986

[35] Schilaty ND Savoldi F Nasr Z Weinshenker BG. Neuromotor control associates with muscle weakness observed with McArdle sign of multiple sclerosis. Ann Clin Transl Neurol. (2022) 9(3):1–14. 10.1002/acn3.51526 PMC8994990 35289110

[36] Franklin DW Wolpert DM. Computational mechanisms of sensorimotor control. Neuron. (2011) 72(3):425–42. 10.1016/j.neuron.2011.10.006 22078503

[37] Gorassini M Yang JF Siu M Bennett DJ. Intrinsic activation of human motoneurons: possible contribution to motor unit excitation. J Neurophysiol. (2002) 87(4):1850–8. 10.1152/jn.00024.2001 11929906

[38] del Vecchio A Casolo A Negro F The increase in muscle force after 4 weeks of strength training is mediated by adaptations in motor unit recruitment and rate coding. J Physiol. (2019) 7:1873–87. 10.1113/JP277250 PMC6441907 30727028

[39] Urbach D Nebelung W Weiler H Awiszus F. Bilateral deficit of voluntary quadriceps muscle activation after unilateral ACL tear. Med Sci Sports Exerc. (1999) 31(12):1691. 10.1097/00005768-199912000-00001 10613416

[40] Strandberg S Lindstrom M Wretling ML Aspelin P Shalabi A. Muscle morphometric effect of anterior cruciate ligament injury measured by computed tomography: aspects on using non-injured leg as control. BMC Musculoskelet Disord. (2013) 14(150):1–9. 23628130 10.1186/1471-2474-14-150 PMC3654923

[41] Harkey MS Luc-Harkey BA Lepley AS Persistent muscle inhibition after ACL reconstruction. Med Sci Sports Exerc. (2016) 1(July. 10.1249/MSS.0000000000001046 27434085

[42] Nagelli Cv Hewett TE. Should return to sport be delayed until 2 years after anterior cruciate ligament reconstruction? Biological and functional considerations. Sports Medicine (2016). 27402457 10.1007/s40279-016-0584-z PMC5226931

[43] Maestroni L Read P Bishop C Turner A. Strength and power training in rehabilitation: underpinning principles and practical strategies to return athletes to high performance. Sports Med. (2020) 50(2):239–52. 10.1007/s40279-019-01195-6 31559567

[44] Paterno Mv Ford KR Myer GD Heyl R Hewett TE. Limb asymmetries in landing and jumping 2 years following anterior cruciate ligament reconstruction. Clin J Sport Med. (2007) 17(4):258–62. 10.1097/JSM.0b013e31804c77ea 17620778

[45] Paterno Mv Huang B Thomas S Hewett TE Schmitt LC. Clinical factors that predict a second ACL injury after ACL reconstruction and return to sport preliminary development of a clinical decision algorithm. Orthop J Sports Med. (2017) 5(12):1–7. 10.1177/2325967117745279 PMC5753959 29318172

[46] Ardern CL Glasgow P Schneiders A 2016 Consensus statement on return to sport from the first world congress in sports physical therapy, Bern. Br J Sports Med. (2016) 50(14):853–64. 10.1136/bjsports-2016-096278 27226389

[47] Hunnicutt JL McLeod MM Slone HS Gregory CM. Quadriceps neuromuscular and physical function after anterior cruciate ligament reconstruction. J Athl Train. (2020) 55(3):238–45. 10.4085/1062-6050-516-18 31995392 PMC7093924

[48] Shelbourne KD Gray T Haro M. Incidence of subsequent injury to either knee within 5 years after anterior cruciate ligament reconstruction with patellar tendon autograft. Am J Sports Med. (2009) 37(2):246–51. 10.1177/0363546508325665 19109531

[49] Nagai T Schilaty ND Laskowski ER Hewett TE. Hop tests can result in higher limb symmetry index values than isokinetic strength and leg press tests in patients following ACL reconstruction. Knee Surg Sports Traumatol Arthrosc. (2020) 28(3):816–22. 10.1007/s00167-019-05513-3 31025059 PMC6814513

